# Construction of environmental risk score beyond standard linear models using machine learning methods: application to metal mixtures, oxidative stress and cardiovascular disease in NHANES

**DOI:** 10.1186/s12940-017-0310-9

**Published:** 2017-09-26

**Authors:** Sung Kyun Park, Zhangchen Zhao, Bhramar Mukherjee

**Affiliations:** 1 Department of Epidemiology, School of Public Health, University of Michigan, 1415 Washington Heights, Ann Arbor, MI 48109 USA; 2 Department of Environmental Health Sciences, School of Public Health, University of Michigan, Ann Arbor, MI USA; 3 Department of Biostatistics, School of Public Health, University of Michigan, Ann Arbor, MI USA

**Keywords:** Bayesian additive regression tree (BART), Bayesian kernel machine regression (BKMR), Cardiovascular disease, Elastic-net, Environmental risk score (ERS), Machine learning, Metals, Mixtures, Multipollutants, Super Learner

## Abstract

**Background:**

There is growing concern of health effects of exposure to pollutant mixtures. We initially proposed an Environmental Risk Score (ERS) as a summary measure to examine the risk of exposure to multi-pollutants in epidemiologic research considering only pollutant main effects. We expand the ERS by consideration of pollutant-pollutant interactions using modern machine learning methods. We illustrate the multi-pollutant approaches to predicting a marker of oxidative stress (gamma-glutamyl transferase (GGT)), a common disease pathway linking environmental exposure and numerous health endpoints.

**Methods:**

We examined 20 metal biomarkers measured in urine or whole blood from 6 cycles of the National Health and Nutrition Examination Survey (NHANES 2003–2004 to 2013–2014, *n* = 9664). We randomly split the data evenly into training and testing sets and constructed ERS’s of metal mixtures for GGT using adaptive elastic-net with main effects and pairwise interactions (AENET-I), Bayesian additive regression tree (BART), Bayesian kernel machine regression (BKMR), and Super Learner in the training set and evaluated their performances in the testing set. We also evaluated the associations between GGT-ERS and cardiovascular endpoints.

**Results:**

ERS based on AENET-I performed better than other approaches in terms of prediction errors in the testing set. Important metals identified in relation to GGT include cadmium (urine), dimethylarsonic acid, monomethylarsonic acid, cobalt, and barium. All ERS’s showed significant associations with systolic and diastolic blood pressure and hypertension. For hypertension, one SD increase in each ERS from AENET-I, BART and SuperLearner were associated with odds ratios of 1.26 (95% CI, 1.15, 1.38), 1.17 (1.09, 1.25), and 1.30 (1.20, 1.40), respectively. ERS’s showed non-significant positive associations with mortality outcomes.

**Conclusions:**

ERS is a useful tool for characterizing cumulative risk from pollutant mixtures, with accounting for statistical challenges such as high degrees of correlations and pollutant-pollutant interactions. ERS constructed for an intermediate marker like GGT is predictive of related disease endpoints.

**Electronic supplementary material:**

The online version of this article (10.1186/s12940-017-0310-9) contains supplementary material, which is available to authorized users.

## Background

Over the last several decades, extensive research on health effects of environmental pollutant exposures has advanced our understanding in pollutant toxicities and related biological mechanisms. These led us to control and lower national regulatory standards for some pollutants (e.g., blood lead levels in children), which has reduced the burden of disease and prevented substantial environmental exposure related diseases. Despite these achievements, a huge research gap remains unanswered: *What is the health effect of exposure to pollutant mixtures*? Although there is growing concern of potential health effects of exposure to pollutant mixtures, most previous studies have been limited to single pollutants, i.e., the unit of analysis is based on a single pollutant. This is due to statistical challenges, such as high degrees of correlation between pollutants, confounding due to co-pollutants (i.e., a spurious association in a single pollutant approach may be observed if the single pollutant is a proxy for other co-pollutants or a mixture of pollutants), lack of replication cohorts, and lack of statistical approaches to evaluating pollutant mixtures [1]. Other methodologic challenges, such as difficulty in exposure assessment of pollutant mixtures with regards to accuracy and feasibility and measurement errors, are also important to hinder multipollutant approaches but the present study will focus on statistical challenges.

Recently, several methods have been proposed to explore health effects of multiple pollutants [2–4]. These include variable selection approaches (least absolute shrinkage and selection operator (LASSO) [5], elastic-net [6], adaptive elastic-net [7]); dimension reduction techniques (principal component analysis (PCA), partial least squares (PLS) [8], weighted quantile sum (WQS) regression [9]); Bayesian approaches (Bayesian model averaging (BMA) [10], Bayesian kernel machine regression (BKMR) [11]); and recursive partitioning (classification and regression tree (CART) [12], random forest [13], Bayesian additive regression tree (BART) [14]). A few studies implemented these methods to analyze multiple pollutants [3, 11, 15–18]. A recent workshop organized by the National Institute of Environmental Health and Sciences (NIEHS) suggested that there is a need for development of novel statistical approaches and appropriate use of available statistical methods in the analysis of combined exposure data from epidemiologic studies [19]. It is also important that methods accounting for the interactions between pollutants within a mixture and cumulative pollutant exposure are needed to estimate the risk of disease [1].

To address these challenges and research gaps, we propose an updated ‘Environmental Risk Score (ERS)’. We initially proposed an ERS as a potential summary measure of effects of multiple pollutants in epidemiologic research [20]. The underlying idea behind an ERS is to build a predictive risk model as a weighted sum of the pollutant levels from simultaneous assessment of multiple pollutants [20]. Weights are determined by the magnitudes (standardized regression coefficients) of the association between each pollutant and the outcome of interest. Our initial study of ERS has several limitations in that quantification of health effects was based on the main effects of individual pollutants; pollutant-pollutant interactions were not considered; and the computed ERS is conceptualized and created in a disease-specific way (i.e., an ERS for one disease is not applicable for another disease) [20]. The updated ERS we propose here will account for these two limitations. The use of modern machine learning methods we consider also bypass the limitation of working with weighted parameters to derive the ERS and offer the flexibility of working directly with predictions that can be generated by a wide class of tools (for example, tree-based methods, smoothing and selection methods) beyond the traditional regression model.

With this objective, we examined the association between metal mixtures and cardiovascular health outcomes in U.S. adults. Heavy metals are widespread, and notable for their toxic effects even at low levels of exposure encountered in the general environment. The cardiovascular system is a main target organ for various heavy metals [21, 22]. Common biological mechanisms by which heavy metals affect the cardiovascular system include increased oxidative stress and lipid peroxidation, inhibition of antioxidant defense systems, smooth muscle cell proliferation, and endothelial injury and apoptosis [22, 23]. Although cardiovascular effects of individual metals have been extensively studied, little is known about cumulative effects of metal mixtures. To test this question, we examined 20 metal biomarkers measured in urine or whole blood from 6 cycles of the National Health and Nutrition Examination Survey (from NHANES 2003–2004 to 2013–2014). We first constructed ERS of metal mixtures for gamma-glutamyl transferase (GGT), a marker of oxidative stress [24, 25], because oxidative stress is a common disease pathway linking environmental pollutant exposure and numerous health endpoints. We then evaluated the associations between ERS and cardiovascular endpoints (blood pressure, hypertension and total and cardiovascular disease (CVD) mortality). Therefore, the constructed ERS will reflect cumulative risk of oxidative stress due to metal mixtures and can also be applied to other health outcomes relevant to oxidative stress, such as cancer, type-2 diabetes, etc. To identify important metals and potential metal-metal interactions, we employed adaptive elastic-net [7], BART [14] and BKMR [11]. We also used Super Learner, an ensemble machine learning prediction algorithm that utilizes a weighted combination of many candidate learners [26]. We compared the prediction performance among these different statistical approaches in a test set to provide insights with regards to data complexity (e.g., sample size, number of predictors, non-linearity, high degrees of correlation, and potential pollutant-pollutant interactions).

## Methods

### Study population

Data were from the U.S. NHANES cycles from 2003 to 2004 through 2013–14 (six continuous NHANES cycles). Two earlier cycles of NHANES (1999–2000 and 2001–2002) were not included because arsenic species were not assessed in those cycles. NHANES is a cross-sectional study designed to be representative of the health and diet of the non-institutionalized U.S. population. NHANES employs a complex, multi-stage sampling design with accompanying design weights. For the present study, 10,805 adults aged 20 years or older who participated in the sub-study of heavy metals in urine, were eligible. We excluded 1141 participants who had missing data in the outcome (GGT, *n* = 663), exposures (heavy metals, *n* = 862) and core covariates (smoking, education, body mass index (BMI), *n* = 197) (the numbers of missing in parentheses are not mutually exclusive), resulting in the sample size of 9664 for ERS construction. Those excluded were older and more likely to be female and non-Hispanic black and less educated (Additional file 1: Table S1). Any potential confounding bias by these factors was minimized by adjustment as covariates in regression models. Given that participants did not know their exposure levels at the recruitment, selection bias due to differential missing data in exposure is unlikely. We additionally excluded 464 and 489 participants when examining blood pressure and hypertension, respectively, due to missing in information on blood pressure and hypertension. For mortality, 6404 participants were available for analysis because mortality data were not available in the last two cycles. NHANES is a publicly available data set and all participants in NHANES provide written informed consent, consistent with approval by the National Center for Health Statistics Institutional Review Board.

### Assessment for heavy metals

Heavy metals in urine (antimony, total arsenic, arsenous acid, arsenic acid, arsenobetaine, arsenocholine, monomethylarsonic acid, dimethylarsonic acid, barium, cadmium, cobalt, cesium, molybdenum, lead, thallium, tungsten, uranium: *n* = 17) and whole blood (lead, cadmium, total mercury: *n* = 3) were analyzed with inductively coupled-plasma dynamic reaction cell-mass spectrometry (ICP-DRC-MS). Detailed laboratory methods and quality control/quality assurance data available in the NHANES website (for example, the laboratory procedure manual for heavy metals in urine in NHANES 2003–2004 available at https://wwwn.cdc.gov/nchs/data/nhanes/2003-2004/labmethods/l06_c_met_hm.pdf). Platinum and beryllium in urine were excluded because more than 95% observations were below limits of detection (LODs). In NHANES, concentrations below LOD were imputed with a value equal to LOD/√2. Although there is some concern in this commonly used substitution method [27], we did not conduct other alternative methods to avoid too complicated modeling and because statistical approaches for mixtures do not support such modeling.

### Assessment for outcomes and covariates

Serum GGT was assayed with Beckman Synchron LX20 or Beckman UniCel DxC800 Synchron via an enzymatic rate method. Blood pressure was measured up to four consecutive times by certified examiners with standardized protocols. We calculated means of systolic and diastolic blood pressure (SBP/DBP) by averaging up to three measures after disregarding the first reading. Hypertension was defined as SBP/DBP ≥ 140/90 mmHg, self-reported physician diagnosis of hypertension, or self-reported use of hypertension medication. Public-use linked mortality data were available for NHANES through 2009–2010. These data provides mortality follow-up data from the date of survey participation through December 31, 2011. Therefore, only the first 4 cycles from 2003 to 2004 to 2009–2010 were used for mortality. Total mortality and cause-specific mortalities (CVD (the 10th International Classification of Disease (ICD-10): I00-I09, I11, I13, I20-51) and cancer (C00-C97)) were linked to NHANES participants. Important covariates were chosen a priori and included age, sex, race/ethnicity (Mexican American, Other Hispanic, non-Hispanic white, non-Hispanic black, Other), education (<high school diploma, high school diploma, and >high school diploma), smoking status (never, former, current), BMI, and urinary creatinine. We selected education as an indicator of socioeconomic status because it is widely used and has less missing data than other proxies, such as household income or poverty income ratio. Urinary creatinine, an indicator of urine dilution [28, 29], was measured using either Jaffe reaction or an enzymatic method.

### Statistical analysis

Although we used complex sampled NHANES data, we did not consider survey components (sampling weights, clusters, and strata) when constructing ERS because most statistical packages for implementing the ML methods do not allow such survey components. We used the survey package in R (version 3.3.1) in the final analyses of associating ERS and cardiovascular outcomes.

**Fig. 1:**
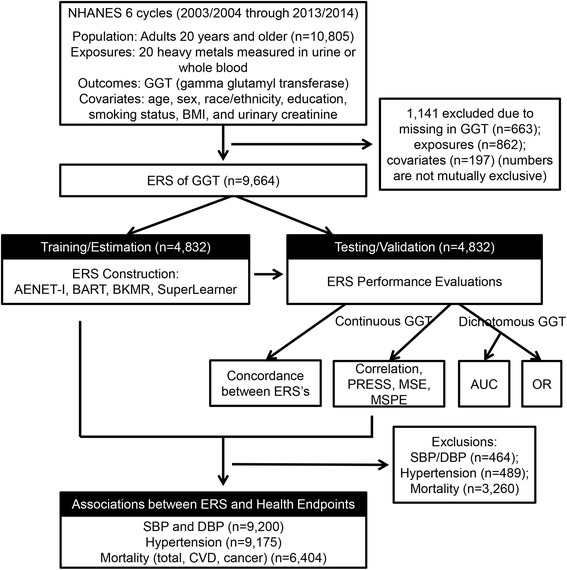
Schematic diagram of Environmental Risk Score (ERS) construction and analytical methods. AENET-I, adaptive elastic-net with main effects and pairwise interactions; BART, Bayesian additive regression tree; BKMR, Bayesian kernel machine regression; PRESS, predicted residual sums of squares; MSE, mean square error; MSPE, mean square prediction error; AUC, area under the receiver operating characteristics curve; OR, odds ratio; SBP/DBP, systolic and diastolic blood pressure; CVD, cardiovascular disease

We calculated pair-wise correlations among 20 metals and created a correlation-matrix heat map. We applied logarithmic transformation with base 10 to GGT and metal pollutants because the distributions of the raw values were highly skewed and the shapes of dose–response relationships were closer to be linear with log-transformation. We chose log with base 10 rather than natural log for easier interpretation of regression coefficients (i.e., one unit increase in a log-transformed variable is equivalent to a ten-fold increase in its raw values). All analyses were conducted using R. A schematic representation of the data accumulation and analytic procedures is presented in Fig. 1.

### Construction of ERS using statistical approaches for pollutant mixtures

We utilized adaptive elastic net, Bayesian Additive Regression Trees (BART), Bayesian Kernel Machine Regression (BKMR), and Super Learner (SL) to determine heavy metals contributing to ERS for GGT.

1. Adaptive elastic net (Zou and Zhang, [7])

Elastic net is a hybrid approach that blends LASSO and ridge regression to overcome the limitation of LASSO on data with highly correlated variables [6]. LASSO and ridge regression are regularized regression techniques that include a penalty term to constrain the size of the estimated coefficients. LASSO shrinks coefficients towards exact zeroes and thus performs variable selection. With highly correlated variables as predictors, LASSO tends to select only one out of these correlated variables and ignore the others. Elastic net also executes variable selection, but it has the opportunity to select a group of non-zero collinear variables. Adaptive elastic net, proposed by Zou and Zhang [7], is an adaptive version of elastic net that not only deals with the collinearity problem over elastic net but satisfies the asymptotic normality assumption that allows us to conduct statistical inference and hypothesis testing by providing large sample standard errors (SEs) and *p*-values. Adaptive weights ensure smaller coefficients are shrunk faster to zero, whereas larger coefficients are penalized less.

For subject *i* (*i* = 1,  … , *N*), let *Y*_*i*_ represent the continuous outcome GGT, ${E}_i^j\ \left(j=1,\dots, p\right)$ be the *j*-th given environmental pollutant, and *Z*_*i*_ (*k* × 1) be the vector of covariates/confounders that the model adjusts for, i.e. age, BMI, urinary creatinine, gender, race/ethnicity, smoking status and education. Let X_*i*_ denote all predictors including *Z*_*i*_, *E*_*i*_ and *E*_*i*_ × *E*_*i*_ **.** Then the estimates from the adaptive elastic net method are defined by

$$ \widehat{\beta}=\left(1+\frac{\lambda_2}{N}\right)\left\{ argmin\left(\sum_{i=1}^N{\left({Y}_i-{X}_i\beta \right)}^2+{\lambda}_1\sum_{j=1}^P{\widehat{\omega}}_j\left|{\beta}_j\right|+{\lambda}_2\sum_{j=1}^P{\beta_j}^2\right)\right\}, $$

where ${\widehat{\omega}}_j$ represents the weight of *j*-th metal pollutant, which can be determined by the beta coefficient from elastic net. These weights can allow coefficients of relatively less important variables (metals) to be shrunk to zero’s more efficiently. Optimal tuning parameters (λ_1_ and λ_2_) were chosen based on 5-fold cross-validations to minimize prediction errors. In our application we chose not to conduct variable selection on *Z*_*i*_ ***,*** and thus we did not penalize the coefficients associated with *Z*_*i*_.

We constructed ERS using the adaptive elastic net with an underlying model considering not only the main effects but all possible combinations of pairwise linear interactions (AENET-I). For comparison purposes, we also ran adaptive elastic net with only main effects (AENET-M), which estimate marginal associations between individual variables within a mixture and the outcome. However, in presence of complex non-linear interaction among the predictors, it is hard to assess the marginal effect of a single pollutant without referencing to the value of the other pollutants. The ‘statistical’ interactions in AENET-I imply departures from additive joint effects. ERS was computed as a weighted sum of the selected non-zero predictors from each model:

$$ {ERS}_i=\sum_{j=1}^P\widehat{\beta_j}{E}_i^j+\sum_{k=1}^{P-1}\sum_{l=k+1}^P\widehat{\beta_{kl}}{E}_i^k{E}_i^l $$

where $\widehat{\beta_j}$ is the coefficient of environmental pollutant predictor *j* and $\widehat{\beta_{kl}}$ is the coefficient of the interaction of environmental pollutant predictors *k* and *l.* Note that most coefficients are zero since these coefficients are shrunk by AENET. All models were adjusted for age, sex, race/ethnicity, education, smoking status, BMI, and urinary creatinine. The R package gcdnet (version 1.0.4) [30] was used to implement adaptive elastic net. The variables with non-zero coefficients were identified as noteworthy/important and their corresponding coefficient estimates and *P*-values were computed.

2) Bayesian Additive Regression Trees (BART) (Chipman et al. [14])

BART is a Bayesian “sum-of-trees” model for ensemble inference from a large number of trees [14]. First, we regress *Y*_*i*_∣*Z*_*i*_ and *E*_*i*_∣*Z*_*i*_, let ${Y}_i^{\ast }$ and ${E}_i^{\ast }$ be the residuals obtained from these regression. Then consider the following model.

$$ {Y}_i^{\ast }=f\left({E}_i^{\ast}\right)+{\epsilon}_i,{\varepsilon}_i\sim N\left(0,{\sigma}^2\right). $$

where *f* is an unknown function and ${Y}_i^{\ast }$ is the residual of GGT adjusted for all the covariates *Z*_*i*_. ${E}_i^{\ast }$ is the residual of all the metal levels adjusted for *Z*. We consider to model *f*(*E* ^∗^) by a sum of *m* regression trees $f\left({E}^{\ast}\right)\approx h\left({E}^{\ast}\right)\equiv {\sum}_{j=1}^m{g}_j\left({E}^{\ast}\right)$ where each *g*_*j*_ denotes the *j*-th regression tree.

A sum-of-trees model is basically an additive model with multiple components. It not only estimates interaction effects, but also takes additive effects into account. Therefore parametric pairwise *E x E* interaction terms were not specifically included in the BART models (also not in BKMR and SL below). The key idea of BART is to implement the sum-of-trees model with a prior that regularizes the fit by keeping the individual tree effects small. At each Markov chain Monte Carlo (MCMC) iteration, we produced a draw from the joint posterior (*f*, *σ*)∣(*Y*, *E*, *Z*) and iterated with Gibbs sampling until the convergence with a default choice of m = 200. BART was implemented with R package BayesTree (version 0.3-1.4) [31]. ERS using BART was constructed as a posterior predicted residual of GGT (we use the posterior mean) for subject *i* after removing the effect of *Z* by

$$ {ERS}_i=\widehat{Y^{\ast }} $$

The variable inclusion proportions were computed in the training set as a measure of the variable importance using the R package bartMachine (version 1.2.3) [32].

3) Bayesian kernel machine regression (BKMR) (Bobb et al. [11])

The main idea of kernel machine regression (KMR) is to flexibly model the relationship between a large number of variables and a particular response variable. Our modeling framework is

$$ {Y}_i=h\left({E}_i\right)+{Z}_i^T\beta +{\varepsilon}_i, $$

where *ε*_*i*_ ~ *N*(0, *σ* ^2^) and *h* is a flexible function of metal pollutants *E*_*i*_, which is characterized through a kernel machine representation. Usually, Gaussian kernel is the default choice, which flexibly captures a wide range of underlying functional forms for *h*, and can be expressed as

$$ K\left({E}_i,{E}_{i\hbox{'}}\right)=\exp \left\{-\sum_{j=1}^P{r}_j{\left({E}_i^j-{E}_{i\hbox{'}}^j\right)}^2\right\} $$

Here *E*_*i*_ and *E*_*i*′_ represent vectors of predictors for two different individuals, and *r*_*j*_ > 0 denotes the tuning parameter that control the smoothness of *h* as a function of the exposure *E*. Intuitively, the kernel function shrinks the predicted response of two individuals with similar exposure profiles toward each other. BKMR is based on KMR and conducts Bayesian inference for the model above [11]. Flat priors for the parameters were chosen in this study. Using MCMC methods, we iterated for 2000 times to ensure the convergence of the method and get the optimal parameter estimates (*r*, *β*). BKMR was implemented with R package bkmr (version 0.2.0) [33]. ERS using BKMR was constructed as a predicted value of GGT for subject *i* by

$$ {ERS}_i=\widehat{h}\left({E}_i\right). $$

The posterior inclusion probabilities of each variable were computed in the training set as a measure of variable importance.

4) Super Learner (SL) (van der Laan et al., [27])

The main idea of SL is to find the optimal prediction by using a combination of predictions from a collection of given algorithms to minimize cross-validation risk [26]. R package SuperLearner (version 2.0.21) [34] was used to predict the outcome (here GGT) through a 10-fold cross-validation. The SL algorithm includes estimations from many candidate learners *L*. In our study, we utilized the following learners: tree based methods (R packages: bartMachine, caret, randomForest), regression based methods (glm, gam, step, glm.interaction), shrinkage (regularized regression) methods (ridge, glmnet), Bagging and Boosting methods (xgboost, ipredbagg) and others (nnls, SVM).

Suppose we consider *M* input algorithms/methods for constructing the ensemble predictor. For a given method *m* (*m* = 1, 2, 3,  … , *M*), we fit a model of the form *Y* ^∗^ = *ψ*_*m*_(*E* ^∗^), where *Y* ^∗^ is the residual of GGT adjusted for all the covariates *Z* and *E* ^∗^ is the residual of all the metal levels after regressing on *Z*. Each method provides a prediction of the form $\widehat{Y_m^{\ast }}=$ ${\widehat{\psi}}_m\left({E}^{\ast}\right)$. We restrict our attention to candidate ensemble predictors of the form

$$ {\psi}_{SL}\left({E}^{\ast}\right)={\sum}_{m=1}^M{a}_m{\psi}_m\left({E}^{\ast}\right)\kern0.5em \mathrm{such}\kern0.5em \mathrm{that}\kern0.5em \mathrm{each}\kern0.5em {a}_m\ge 0,\forall m\kern0.5em \mathrm{and}\kern0.5em {\sum}_{m=1}^M{a}_m=1. $$

The set of weights corresponding to each method *a* = {*a*_*m*_, *m* = 1⋯, *M*} is now determined by a V-fold cross-validation. For each of the *v* = 1 ,  ⋯  , *V* fold, the entire learning set of size *n* is divided into binary splits of training and validation set that are mutually exclusive. For the *v-*th fold, we use the indicator (${\mathrm{B}}_n^v(i)=0$) to denote observations in the training set and $\Big({\mathrm{B}}_n^v(i)=1$) to denote observations in the validation set. Let *n* ^*test*^(*v*) denote the size of the test or validation sample in the *v*-th fold. The vector *a* is obtained by estimating ${\widehat{\psi}}_m$ on the training data but evaluating the risk on the test data and minimizing the V-fold cross-validated prediction loss.

$$ \widehat{a}=\mathit{\arg}\kern0.5em {\mathrm{min}}_a{\sum}_v\frac{1}{n^{test}(v)}{\sum}_{i:{B}_n^v(i)=1}{\left({Y}_i^{\ast }-{\sum}_{m=1}^M{a}_m{\widehat{\psi}}_m\left({E}_i^{\ast}\right)\right)}^2. $$

The final SL fit, i.e.,ERS_*SL*_is computed by combining $\widehat{a}$ with ${\widehat{\psi}}_m\left({E}^{\ast}\right)$, *m* = 1 , 2 ,  …  , *M*:

$$ {\mathrm{ERS}}_{SL}={\sum}_{m=1}^M\widehat{a_m}{\widehat{\psi}}_m\left({E}^{\ast}\right). $$

There is no single consensus measure to evaluate variable importance in the SL. We therefore, computed a sensible metric in the training set as done in BART and BKMR. First we ran SL with all 20 metals and calculated the sum of squared errors (SSE). We then removed one metal, ran SL with the remaining 19 metals and calculated SSE [SSE(−i)]. We repeated this for each metal. The variable importance was computed as the difference between SSE(−i) and SSE divided by SSE [i.e., (SSE(−i) – SSE)/SSE]. For comparison purposes, we also compared individual algorithms within SL in terms of the model prediction performance.

### Assessment of predictive power of ERS

To evaluate and compare the performance of 5 ERS’s (AENET-I, AENET-M, BART, BKMR, and SL), we randomly split the full data (all cycles combined) by a 1∶1 ratio: the first part (*n*  =  4832) used for estimation/training and the second part (*n*  =  4832) for validation/testing. We repeated this random split three times and constructed ERS each time but the results (i.e., selected predictors or variable importance) were consistent. We, therefore, report the results based on the first split data. Three metrics were computed: First, we used linear regression for GGT and fit ERS as a continuous variable and computed correlation coefficients between GGT and ERS and the predicted residual sums of squares (PRESS), a statistic measuring model goodness of fit. Mean squared error (MSE) in the training data and mean squared prediction error (MSPE) in the testing data were also calculated to assess prediction performance. Second, we dichotomized GGT at the 90th percentile (50 U/L), and conducted logistic regression analysis with this dichotomized outcome and continuous ERS as predictor. We used area under the receiver operating characteristic (ROC) curve or AUC to assess predictive ability of the ERS with these binary endpoints. Third, in order to assess risk stratification/discrimination power of the ERS, we further categorized ERS by its quintiles based on the distribution in the training set and conducted logistic regression with categorical ERS in the testing set. We computed the odds ratio (OR) for the highest quintile vs. the lowest quintile of ERS to measure the risk stratification properties of ERS.

### Associations between ERS and cardiovascular endpoints

Next, we examined the associations between each ERS and cardiovascular endpoints, such as SBP, DBP, hypertension, and mortality (total and cardiovascular). Survey linear regression, survey logistic regression and survey Cox-proportional hazard models were used for SBP/DBP, hypertension, and mortality, respectively. Age was used as the time scale in Cox-proportional hazard models [35]. Using age as the time-scale implies delayed entry with left truncation occurring at the age at inclusion. In this approach, the hazard function can be directly interpreted as the age-specific incident function. We also examined cancer mortality which is a non-cardiovascular endpoint but related to oxidative stress as a potential biological mechanism. Separate Cox models were fit for each cause-specific outcome and log(hazard ratio (HR)) was obtained from each Cox model. The same covariates used above were adjusted for all models. To standardize the distributions of different ERS’s, we computed z-scores for each ERS by subtracting the mean of the corresponding ERS divided by its standard deviation (SD). We report β coefficients (95% confidence intervals (CIs)) for SBP/DBP, ORs for hypertension, and HRs for mortality outcomes for a one-unit increase in the z-score of ERS which is equivalent to a one SD increase in its original scale.

## Results

Table 1 presents population characteristics for the whole and by each cycle. Mean (SD) of age was 49.2 (17.2) years and approximately 51% were female. Overall, the proportions of Mexican American, other Hispanic, Non-Hispanic white, Non-Hispanic black, and other race/ethnicity were 16.7%, 8.1%, 46.6%, 20.4%, and 8.2%, respectively. Starting in the cycle of 2007–2008, all Hispanics were oversampled, not just Mexican Americans, thus the proportion of other Hispanic increased since this cycle. Likewise, Asian Americans were oversampled starting in the cycle of 2011–2012. There were decreasing trends in the prevalence of current smokers and low education attainment (<high school diploma) over the study period. The means (SDs) of GGT, SBP and DBP were 21.7 (1.94) U/L, 123.2 (18.7) mm Hg, and 70.0 (12.0) mm Hg, respectively. The prevalence of hypertension was 36.9%. Over the mean of 4.5 years of follow-up, the mortality rates of all causes, CVD and cancer were 5.6%, 1.5% and 1.4%, respectively.

**Table 1:** Characteristics of the study population overall and by NHANES cycle

	Cycle						Overall
	2003-2004	2005-2006	2007-2008	2009-2010	2011–2012	2013-2014	
	*N* = 1443	*N* = 1418	*N* = 1664	*N* = 1884	*N* = 1557	*N* = 1698	N = 9664
CONTINUOUS, mean(SD)							
Age, years	51.0 (19.5)	48.3 (18.8)	50.2 (17.6)	49.4 (17.8)	48.0 (17.7)	48.3 (17.2)	49.2 (17.2)
BMI, kg/m^2^	28.6 (6.32)	28.7 (6.70)	28.7 (6.13)	29.1 (6.86)	28.7 (6.90)	29.1 (7.11)	28.8 (6.69)
GGT, U/L	21.5 (1.91)	20.85 (1.96)	24.1 (1.92)	22.1 (1.92)	20.5 (1.92)	21.3 (1.96)	21.7 (1.94)
SBP, mm Hg	126.0 (20.7)	123.0 (19.3)	123.7 (18.6)	121.9 (18.4)	122.5 (17.8)	122.6 (17.5)	123.2 (18.7)
DBP, mm Hg	70.3 (12.3)	69.1 (12.7)	70.4 (11.8)	69.0 (12.2)	71.0 (11.8)	70.2 (11.3)	70.0 (12.0)
CATEGORICAL, N (%)							
Female	839 (52.08)	825 (51.79)	966 (50.31)	1078 (52.00)	871 (49.77)	961 (51.72)	4911 (50.82)
Race/Ethnicity							
Mexican American	323 (20.05)	311 (19.52)	322 (16.77)	384 (18.52)	154 (8.80)	267 (14.37)	1618 (16.74)
Other Hispanic	39 (2.42)	47 (2.95)	234 (12.19)	217 (10.47)	179 (10.23)	159 (8.56)	779 (8.06)
Non-Hispanic White	852 (52.89)	816 (51.22)	893 (46.51)	973 (46.94)	639 (36.51)	805 (43.33)	4501 (46.57)
Non-Hispanic Black	341 (21.17)	363 (22.79)	402 (20.94)	372 (17.95)	458 (26.17)	370 (19.91)	1976 (20.45)
Other	56 (3.48)	56 (3.52)	69 (3.59)	127 (6.13)	320 (18.29)	257 (13.83)	790 (8.17)
Smoking Status							
Never	830 (51.55)	843 (52.92)	1015 (52.89)	1116 (53.84)	1002 (57.29)	1044 (56.19)	5242 (54.24)
Former	337 (20.93)	338 (21.22)	428 (22.30)	444 (21.42)	340 (19.44)	376 (20.24)	2000 (20.70)
Current	443 (27.52)	412 (25.86)	476 (24.80)	513 (24.75)	407 (23.27)	438 (23.57)	2422 (25.06)
Education							
< High School	486 (30.25)	450 (28.28)	611 (31.82)	591 (28.55)	420 (24.03)	393 (21.16)	2599 (26.89)
High School	850 (52.89)	836 (52.54)	966 (50.32)	1082 (52.27)	872 (49.89)	996 (53.64)	5018 (51.92)
College or Above	271 (16.86)	305 (19.17)	343 (17.86)	397 (19.18)	456 (26.09)	468 (25.20)	2047 (21.18)
Hypertension	549 (40.76)	450 (33.06)	608 (37.98)	666 (36.78)	550 (36.74)	595 (36.28)	3418 (36.92)
Mortality							
Total	163 (11.32)	102 (7.19)	62 (3.73)	32 (1.70)	NA	NA	359 (5.61)
CVD	42 (2.92)	33 (2.33)	17 (1.02)	2 (0.11)	NA	NA	94 (1.47)
Cancer	38 (2.64)	25 (1.76)	14 (0.84)	13 (0.69)	NA	NA	90 (1.41)

*BMI* body mass index, *GGT* gamma-glutamyl transferase, *SBP* systolic blood pressure, *DBP* diastolic blood pressure, *CVD* cardiovascular disease

**Fig. 2:**
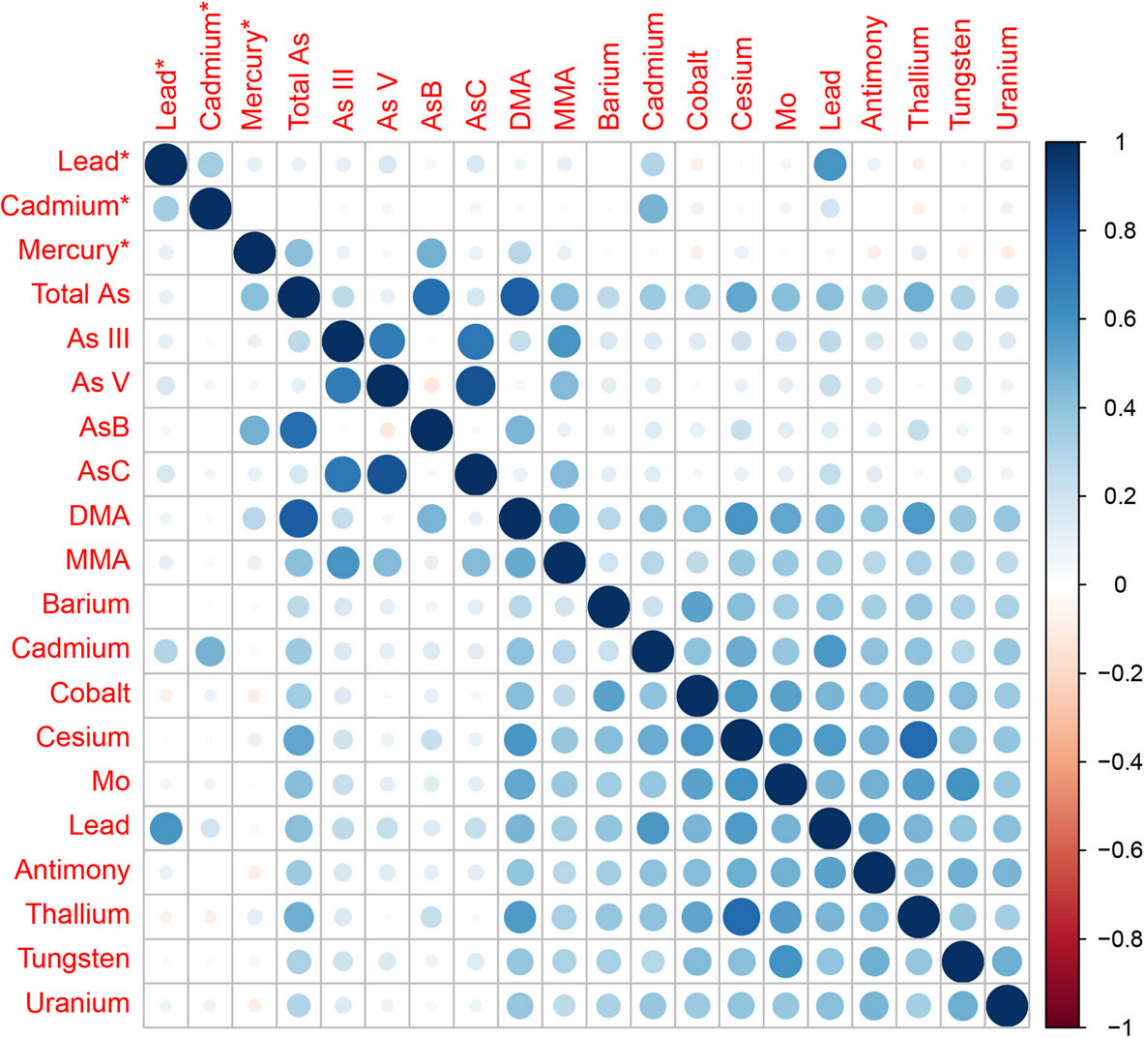
Heat map of Spearman correlations between metal biomarkers. Asterisk next to the metal names indicates metals measured in whole blood. As, arsenic; As III, arsenous acid; As V, arsenic acid; MMA, monomethylarsonic acid (MMA); DMA, dimethylarsonic acid; Mo, molybdenum

Table 2 shows geometric means (GMs) and geometric standard deviations (GSDs) of metals measured in either whole blood or urine overall and by each cycle. Most metals showed that GMs were higher than GSDs, suggesting skewness. There were decreasing trends in the population-level concentrations of lead in both blood and urine. Urinary arsenobetaine concentrations increased in the cycles of 2011–2012 and 2013–2014, which may result from the increased proportion of Asians who eat more fish (arsenobetaine is mainly from fish consumption). Figure 2 shows a heat map of Spearman correlations between metal biomarkers. There were moderate to high correlations between blood lead and blood cadmium (Spearman correlation (rho) = 0.35); between blood total mercury and urinary arsenobetain (rho = 0.47); and among arsenic species (monomethylarsonic acid (MMA), arsenous acid (As III), arsenic acid (As V), and arsenocholine: rho = 0.43 to 0.87). A group of urinary metals including total arsenic, dimethylarsonic acid (DMA), cadmium, lead, cobalt, thallium, cesium, barium, uranium, tungsten, and molybdenum also had modest to high correlations each other (rho = 0.22 to 0.82).

**Table 2:** Geometric means and geometric standard deviations of metals overall and by NHANES cycle

	Cycle						Overall *N* = 9664
	2003-2004	2005-2006	2007-2008	2009-2010	2011–2012	2013-2014	
	*N* = 1443	*N* = 1418	*N* = 1664	*N* = 1884	*N* = 1557	*N* = 1698	
In whole blood							
Lead, μg/dL	1.67 (1.92)	1.47 (2.01)	1.49 (1.91)	1.31 (1.95)	1.13 (2.01)	1.01 (1.97)	1.32 (2.00)
Cadmium, μg/L	0.39 (2.27)	0.37 (2.17)	0.39 (2.15)	0.38 (2.13)	0.36 (2.27)	0.33 (2.30)	0.37 (2.22)
Total Mercury, μg/L	0.88 (2.78)	0.96 (2.54)	0.90 (2.58)	1.01 (2.59)	0.94 (2.87)	0.87 (2.70)	0.93 (2.68)
In urine, μg/L							
Antimony	0.08 (1.81)	0.07 (2.29)	0.06 (2.15)	0.06 (2.22)	0.05 (2.00)	0.04 (2.36)	0.06 (2.21)
Total Arsenic	8.92 (3.13)	9.91 (3.21)	8.77 (2.99)	10.07 (3.25)	8.81 (3.29)	7.14 (3.06)	8.88 (3.17)
Arsenous acid	0.83 (1.21)	0.87 (1.18)	0.87 (1.21)	0.88 (1.24)	0.45 (1.63)	0.32 (2.84)	0.65 (1.91)
Arsenic acid	0.73 (1.18)	0.73 (1.21)	0.72 (1.17)	0.73 (1.19)	0.64 (1.21)	0.57 (1.13)	0.68 (1.21)
Arsenobetaine	1.77 (5.15)	2.17 (5.81)	1.45 (5.55)	1.82 (6.17)	2.75 (4.31)	2.27 (3.85)	1.99 (5.19)
Arsenocholine	0.41 (1.18)	0.43 (1.21)	0.43 (1.19)	0.44 (1.35)	0.21 (1.41)	0.10 (1.78)	0.30 (1.91)
Dimethylarsonic acid	3.84 (2.20)	4.01 (2.18)	3.88 (2.19)	3.87 (2.39)	4.14 (2.41)	3.46 (2.19)	3.85 (2.27)
Monomethylacrsonic acid	0.82 (1.65)	0.85 (1.56)	0.85 (1.56)	0.83 (1.60)	0.79 (1.55)	0.42 (2.41)	0.73 (1.85)
Barium	1.29 (2.64)	1.33 (2.80)	1.28 (2.71)	1.30 (2.59)	1.07 (2.69)	0.97 (2.79)	1.20 (2.72)
Cadmium	0.29 (2.69)	0.26 (2.74)	0.27 (2.65)	0.25 (2.62)	0.22 (2.78)	0.18 (2.94)	0.24 (2.77)
Cobalt	0.31 (2.24)	0.37 (2.27)	0.35 (2.16)	0.34 (2.28)	0.31 (2.29)	0.37 (2.27)	0.34 (2.26)
Cesium	4.54 (2.08)	4.63 (2.02)	4.38 (1.97)	4.11 (1.94)	3.89 (2.00)	3.92 (1.99)	4.22 (2.00)
Lead	0.70 (2.15)	0.65 (2.43)	0.57 (2.34)	0.53 (2.33)	0.41 (2.45)	0.32 (2.51)	0.51 (2.47)
Molybdenum	38.27 (2.42)	42.97 (2.33)	42.17 (2.43)	40.46 (2.37)	36.95 (2.41)	32.42 (2.47)	38.64 (2.42)
Thallium	0.14 (2.12)	0.15 (2.06)	0.14 (2.06)	0.14 (2.05)	0.15 (2.07)	0.14 (2.14)	0.14 (2.09)
Tungsten	0.06 (2.61)	0.08 (2.74)	0.09 (2.76)	0.07 (2.67)	0.07 (2.69)	0.05 (2.95)	0.07 (2.77)
Uranium	0.01 (2.23)	0.01 (2.63)	0.01 (2.75)	0.01 (2.70)	0.01 (2.44)	0.01 (2.97)	0.01 (2.65)

**Fig. 3:**
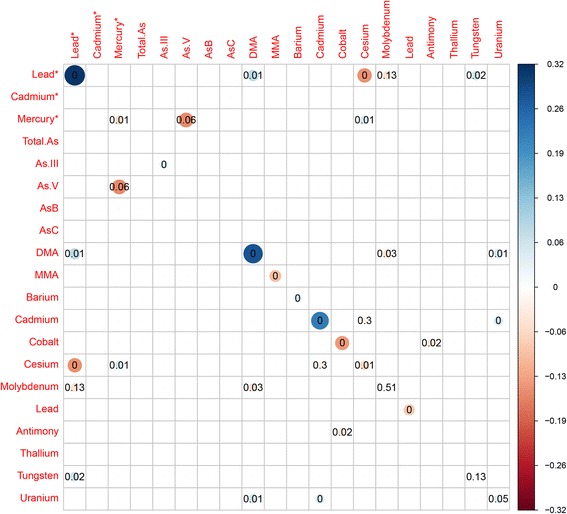
Selected predictors of the main effects (diagonal cells) and pairwise interactions (off-diagonal combinations) for serum gamma-glutamyl transferase (GGT) in adaptive elastic net. Bubble size indicates the magnitude of the association. The number inside indicates p-value. Asterisk next to the metal names indicates metals measured in whole blood. As, arsenic; As III, arsenous acid; As V, arsenic acid; MMA, monomethylarsonic acid (MMA); DMA, dimethylarsonic acid; Mo, molybdenum

Figure 3 and Additional file 1: Table S2 shows the variable selection results for AENET-M and AENET-I. Nine predictors (main effects) were selected in AENET-M and 24 predictors (13 main effects and 11 pairwise interactions) were selected in AENET-I. In AENET-I, important metals selected include lead (blood) (β = 0.31, *p* = 6.2 × 10^−6^), cadmium (urine) (β = 0.22, *p* = 9.8 × 10^−7^), DMA (β = 0.28, *p* = 5.9 × 10^−5^), cobalt (β = −0.14, *p* = 1.2 × 10^−8^), MMA (β = −0.10, *p* = 1.3 × 10^−10^), and barium (β = 0.05, *p* = 7.9 × 10^−10^) in terms of either a large magnitude of the association (|β| > 0.1) or a small *p*-value (<1 × 10^−5^). Interactions of cesium-lead (blood) (β = −0.15, *p* = 4.5 × 10^−4^) and of mercury-As V (β = −0.15, *p* = 6.1 × 10^−2^) were also identified. In BART, urinary cadmium seems to contribute to prediction of ERS most (variable importance (the relative proportion of the contribution) = 9.49%) followed by cobalt (7.21%), DMA (7.14%), tungsten (7.13%), MMA (6.78%) and so on (Additional file 1: Table S3). In SL, As V had the highest variable importance measure (25.1%) followed by MMA (24.2%), tungsten (22.9%), total As (22.6%), DMA (22.6%), and cadmium (21.5%) (Additional file 1: Table S3). Combining the ranks of variable importance between BART and SL, cadmium, tungsten, MMA, DMA, As V, barium and cobalt were ranked as the top 7 metals (Additional file 1: Table S3). In BKMR, almost all metals had posterior inclusion probabilities (PIP) of 1 except arsenous acid (As III, PIP = 0.96) and arsenic acid (As V, PIP = 0.85) (data not shown).

The ERS’s from each statistical approach ranged from −0.22 to 0.25 for AENET-M; −0.26 to 0.49 for AENET-I; −0.22 to 0.45 for BART; −1.01 to 1.83 for BKMR; and −0.18 to 0.27 for SL (Table 3). ERS from BKMR had a wider range whereas ERS from SL had a narrower range than other approaches. Pairwise correlations among AENET-I, BART and SL were relatively high (>0.6), whereas BKMR had weak correlations with other approach (<0.12) (Additional file 1: Figure S1).

**Table 3:** Comparison of ERS distribution and risk prediction performance by different statistical approaches

	Base Model^a^	AENET-M	AENET-I	BART	BKMR	SL	Full Model^b^
Distributions of ERS							
Training Set							
Mean (SD)	–	0.00 (0.05)	0.00 (0.06)	0.00 (0.06)	0.00 (0.27)	0.00 (0.06)	–
Range	–	(−0.18, 0.25)	(−0.26, 0.49)	(−0.22, 0.45)	(−1.01, 1.83)	(−0.18, 0.27)	–
Testing Set							
Mean (SD)	–	0.00 (0.05)	0.00 (0.06)	0.00 (0.05)	0.01 (0.08)	0.00 (0.04)	–
Range	–	(−0.22, 0.19)	(−0.22, 0.35)	(−0.22, 0.32)	(−0.34, 0.66)	(−0.17, 0.24)	–
Risk Prediction Performance							
Continuous GGT^c^							
Training Set							
Correlation^d^	–	0.22	0.24	0.35	0.82	0.75	–
MSE	7.2E-02	7.0E-02	6.9E-02	6.4E-02	2.6E-04	3.6E-02	6.7E-02
Testing Set							
Correlation^d^	–	0.25	0.27	0.20	0.00	0.26	–
PRESS	332.9	320.6	316.1	325.1	332.3	321.7	327.2
MSPE	6.9E-02	6.6E-02	6.5E-02	6.7E-02	6.9E-02	6.6E-02	6.8E-02
Dichotomous GGT^e^							
Training Set							
AUC	0.67	0.70	0.71^*^	0.75^†^	>0.99^‡^	0.92^‡^	0.73^†^
95% CI	(0.64, 0.69)	(0.67, 0.72)	(0.68, 0.73)	(0.73, 0.78)	(0.99, 1.00)	(0.91, 0.93)	(0.70, 0.75)
Testing Set							
AUC	0.66	0.69	0.70^*^	0.69	0.66	0.70^*^	0.68
95% CI	(0.64, 0.68)	(0.67, 0.71)	(0.68, 0.72)	(0.66, 0.71)	(0.64, 0.68)	(0.67, 0.72)	(0.66, 0.70)

*AENET-M* adaptive elastic net for main effects, *AENET-I* adaptive elastic net for main effects and pairwise interactions, *BART* Bayesian Additive Regression Tree, *BKMR* Bayesian Kernel Machine Regression, *SL* Super Learner, *GGT* gamma-glutamyl transferase, *MSE* mean square error, *PRESS* predicted residual sums of squares, *MSPE* mean square prediction error, *AUC* area under the receiver operating characteristic curve

^a^Base model contains only covariates (age, sex, race/ethnicity, smoking status, education, body mass index, urinary creatinine)

^b^Full model contains all covariates, main effects and all possible pairwise interactions of metals

^c^GGT was logarithmically transformed. Mean (SD) of log(GGT) = 0.27 (0.21)

^d^Correlation between GGT and ERS

^e^GGT was dichotomized at the 90th percentile (50 I/U)

^*^ *P* < 0.1, ^†^ *P* < 0.05, ^‡^ *P* < 0.01. *P*-values were computed with permutation tests comparing with AUC of the base model

For risk prediction performance for continuous GGT, AENET-I outperformed other approaches in terms of correlation between GGT and ERS, PRESS, and MSPE in the testing set (Table 3). The correlation coefficients between GGT and ERS in the training set were 0.22 in AENET-M and 0.24 in AENET-I and similar and slightly improved correlation coefficients were obtained in the testing set. The correlation coefficient between GGT and ERS in BART was 0.35 in the training set and 0.20 in the testing set. BKMR and SL overfit GGT prediction in the training set (correlation coefficient = 0.82 in BKMR and 0.75 in SL). BKMR poorly predicted GGT in the testing set (correlation coefficient = 0.0) but SL performed reasonably in the testing set (correlation coefficient = 0.20). For dichotomous GGT in the testing set, AENET-I and SL performed best (Table 3 and Additional file 1: Figure S2). The addition of their ERS modestly improved the AUC for GGT (AUC from 0.66 to 0.70 for both). Again, the same overfitting issue was observed for the ERS from BKMR in the training set.

**Fig. 4:**
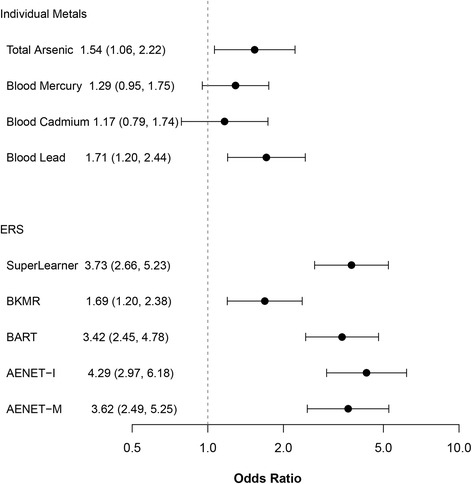
Odds ratios (95% confidence intervals) of having high GGT (50 U/L and above) comparing the highest vs. the lowest quintiles of ERS and individual pollutants that compose the ERS in the testing set. All models were adjusted for age, BMI, creatinine, gender, race/ethnicity, smoking status and education

Figure 4 shows ORs of having high GGT levels (50 U/L) comparing the highest vs. the lowest quintiles of ERS. After controlling for base covariates, ORs of having high GGT levels ranged from 1.69 (95% CI, 1.20 to 2.38) for BKMR, 3.42 (95% CI, 2.45 to 4.78) for BART, 3.62 (95% CI, 2.49 to 5.25) for AENET-M, 3.73 (95% CI, 2.66 to 5.23) for SL, to 4.29 (95% CI, 2.97 to 6.18) for AENET-I. These ORs were even stronger than those for individual metals. ORs comparing the highest vs. the lowest quintile of lead, cadmium, mercury and total arsenic were 1.71 (95% CI, 1.20 to 2.44), 1.17 (95% CI, 0.79 to 1.74), 1.29 (95% CI, 0.95 to 1.75), 1.54 (95% CI, 1.06 to 2.22), respectively. Furthermore, the ERS effect sizes (except ERS for BKMR) were stronger than those for socio-demographic factors or BMI. For example, OR for females vs males was 2.16 (95% CI 1.74, 2.67); OR for 10 kg/m^2^ increase in BMI was 1.44 (95 CI, 1.25, 1.65); and OR for <high school vs. college or higher was 1.76 (95% CI, 1.26, 2.46) (data not shown).

Table 4 presents the associations between ERS and various cardiovascular endpoints. Because the ERS prediction performance for BKMR was not good, we excluded the BKMR-based ERS in these analyses. All ERS’s showed significant associations with systolic and diastolic blood pressure and hypertension. ERS based on SL showed the largest associations: one SD increase in SL-ERS was associated with a 1.03 mmHg (95% CI, 0.57, 1.48) higher in SBP; a 1.61 mmHg (95% CI, 1.28, 1.95) higher in DBP. For hypertension, one SD increase in each ERS from AENET-I, BART and SL were associated with ORs of 1.26 (95% CI, 1.15, 1.38), 1.17 (1.09, 1.25), and 1.30 (1.20, 1.40), respectively. For comparison, blood lead and blood cadmium were associated with ORs of 1.08 (0.99, 1.18) and 1.06 (0.98, 1.16). ERS’s showed non-significant positive associations with both total and CVD mortality. Positive associations with cancer mortality were observed for all ERS’s but those associations did not reach statistical significance except AENET-M (HR = 1.50, 95% CI, 1.05, 2.15).

**Table 4:** Associations of health endpoints (blood pressure, hypertension, and mortality) with ERS’s from different statistical approaches. For the comparison purpose, associations with blood lead and blood cadmium are presented

		AENET-M	AENET-I	BART	SL	Blood Lead	Blood Cadmium
SBP	β (95% CI)	0.03 (−0.45, 0.51)	0.69 (0.20, 1.18)	0.52 (0.07, 0.97)	1.03 (0.57, 1.48)	0.78 (0.22, 1.34)	0.28 (−0.25, 0.82)
DBP	β (95% CI)	1.12 (0.73, 1.50)	1.50 (1.11, 1.88)	0.90 (0.55, 1.24)	1.61 (1.28, 1.95)	0.97 (0.60, 1.34)	0.29 (−0.12, 0.71)
Hypertension	OR (95% CI)	1.11 (1.02, 1.22)	1.26 (1.15, 1.38)	1.17 (1.09, 1.25)	1.30 (1.20, 1.40)	1.08 (0.99, 1.18)	1.06 (0.98, 1.16)
Total mortality	HR (95% CI)	1.07 (0.89, 1.30)	1.07 (0.92, 1.24)	1.07 (0.96, 1.19)	1.15 (0.98, 1.35)	1.08 (0.86, 1.36)	1.37 (1.10, 1.70)
CVD mortality	HR (95% CI)	0.99 (0.69, 1.41)	1.09 (0.78, 1.54)	1.07 (0.76, 2.51)	0.98 (0.71, 1.36)	0.92 (0.60, 1.41)	1.24 (0.80, 1.92)
Cancer mortality	HR (95% CI)	1.50 (1.05, 2.15)	1.24 (0.93, 1.63)	1.23 (0.99, 1.54)	1.23 (0.94, 1.60)	1.41 (1.01, 1.96)	1.50 (1.07, 2.10)

*AENET-M* adaptive elastic net for main effects, *AENET-I* adaptive elastic net for main effects and pairwise interactions, *BART* Bayesian Additive Regression Tree, *SL* Super Learner

Effect estimates (β, odds ratio (OR), and hazard ratio (HR)) are based on a standardized increment which is equivalent to one standard deviation increase in each ERS. All models were adjusted for age (except mortality outcomes), sex, race/ethnicity, body mass index, smoking status, education

## Discussion

There is growing interest in evaluating health effects of real-life environmental exposure as mixtures. Scientists have recognized the need to develop novel statistical approaches to predicting disease risk associated with exposure to pollutant mixtures [19]. The present study proposes an updated ERS [20] using cross-cutting statistical approaches developed to handle statistical challenges in pollutant mixtures: high degrees of correlations among pollutants; pollutant-pollutant interactions; and cumulative risk from pollutant mixtures [1]. Among the ERS’s constructed to predict GGT, an indicator of oxidative stress, AENET-I, a regularized regression-based variable selection method that considered both main effects and pairwise interactions, performed best. Despite their slight worse performance in prediction, BART, a nonparametric Bayesian regression approach that uses dimensionally adaptive random basis elements [14], and SL, an ensemble of multiple learners [26], in which evaluation of interaction effects are built, showed comparable prediction performance only with the main effect terms in their models. The observed associations between ERS and GGT were larger than those for known toxic metals assessed individually, suggesting that cumulative (combined) effects may be larger than each individual effect. We also found significant associations between ERS and DBP and hypertension, implying that individuals who have higher metal mixtures-related oxidative stress may be at higher risk for high blood pressure.

Choice of statistical approaches is a critical step in research on multi-pollutants and mixtures. As listed in Introduction, a number of different statistical approaches have been developed to analyze high dimensional and correlated data. A workshop by NIEHS in 2015, entitled “Statistical Approaches for Assessing Health Effects of Environmental Chemical Mixtures in Epidemiology Studies”, reported that no one statistical approach performs better than another and a statistical approach should be chosen based on a specific scientific question and hypothesis related to pollutant mixtures [19]. Our primary goal and specific question when constructing ERS using 4 different statistical approaches was to develop better prediction models for early health effects on a common disease pathway (i.e., oxidative stress) while accounting for high degree correlations and potential interactions among metals. Then these prediction models are used to assess their risk stratification and discrimination power for predicting specific health endpoints. The statistical approaches we used and other approaches listed in Introduction are ‘agnostic’ and the selected predictors do not necessarily reflect causal agents or biological interactions [36]. However, when predictive modeling is the primary goal rather than causal explanatory modeling, causality of the selected predictors may be of less importance if highly correlated pollutants are examined [37]. Either causal predictors or their correlated proxies should have similar predictive power and in general, variables with less noise are likely to be selected [38]. Biologically-based dose–response functions, such as physiologically based pharmacokinetic (PBPK) modeling, may better capture causal explanation but require more complex modeling that is hard to test and toxicological data that are often not available. Our predictive modeling can provide a good approximation of underlying causal relationships by capturing complex relationships and patterns among pollutants within mixtures [39].

In terms of choice of statistical models, BKMR was found to be the least “scalable” among the methods we considered. AENET-I has the advantage of providing important variables and working in a parametric regression structure and works well under sparsity and large-*p-*small-*n* situation. BART and BKMR are non-parametric methods based on kernel smoothing and recursive partitioning but have difficulty with a truly ultra-dimensional covariate space. SL is a highly flexible and powerful method that can incorporate any prediction generating tool as an ingredient and data-adaptively weigh the predictions. The limitation is that it does not provide measures of variable importance or carry out variable selection. All four methods can handle modest to strong correlation among predictors. While we have allowed our construction of ERS to be driven by statistical considerations, it may be worthwhile to group the pollutants by known biological mechanisms. Bayesian methods and grouped-LASSO type methods have the potential of incorporating this information through a priori grouping and incorporation of priors. Contrasting data-driven methods with biology-driven methods and combining epidemiology with biology in an optimal way remain a relevant research topic.

AENET most likely provides biased coefficients because of shrinkage estimation. Some studies attempted to obtain unpenalized coefficients by re-fitting multiple-exposure OLS regression models with selected variables post E-net selection [17]. In general, it is well-known that running OLS post model selection with the selected variables, using the same dataset leads to high false discovery rates and over-optimistic results. Thus we refrain from using ERS that is constructed by fitting OLS post model-selection. Fitting OLS on a new independent dataset with the selected variables is a valid strategy. There are new variants of LASSO estimates, for example the debiased LASSO that can handle this issue better but are still in developing stages of implementation. With advances in post-model selection theory, we would have better inferential tools under penalized variable selection methods like AENET [40].

One important approach we attempted is to use an oxidative stress marker as the outcome. A challenge for constructing the cumulative effects of multiple pollutant exposure like ERS is that they are disease-specific. ERS constructed to predict CVD may not predict cancer risk well. ERS for common disease pathways, such as oxidative stress, inflammation, epigenetic modification, and endocrine disruption, may capture cumulative early biological effects and discriminate individuals who are at increased risk of manifesting various downstream clinical diseases. GGT has been suggested an oxidative stress marker [24, 25] and widely used in large-scale epidemiologic research [41–43]. Markers that directly measure oxidative damages in vivo such as F2-isoprostane [44, 45] may improve risk prediction and thus better discriminate metal mixture health risk. Our analyses of linking ERS for oxidative stress and clinical outcomes suggest that this cumulative risk score is a useful tool to predict the clinical outcomes related to oxidative stress. One limitation is that given its non-specificity, the ERS in the current study may not capture disease risk for complex diseases as good as the ERS constructed for a specific disease. A more targeted ERS for a specific disease will need to be built with different weights.

Cardiovascular effects of heavy metals, especially lead, cadmium and arsenic, have long been acknowledged and examined in epidemiologic research. A recent expert review by the National Toxicology Program found sufficient evidence to conclude that lead exposure, even at low exposure (<10 μg/dL), is associated with elevated blood pressure and hypertension but the evidence is limited or inadequate for other cardiovascular endpoints [46]. For arsenic, several systematic reviews suggest there is evidence for a causal association between high long-term arsenic exposure and CVD endpoints; however, conclusive evidence is lacking for an association with low-to-moderate arsenic levels [47, 48]. For cadmium, chronic exposure may be an independent risk factor for CVD, but further studies with individual level exposure and standardized CVD outcome assessments and accounting for confounding by smoking are needed to establish a causal association [49]. For other non-essential metals, only a few epidemiologic studies were conducted in relation to CVD [50–54]. A recent systematic review concluded that the current evidence is insufficient to support the causality of the association between environmental metals and CVD because of the small number of studies [55].

Although individual metal effects were not our primary interest, the metals identified in the multipollutant models deserve further consideration. Our multipollutant models (AENET-I, BART and SL) confirmed that those historically established metals (lead, cadmium and arsenic as methylated forms (MMA and DMA)) are important predictors. Interestingly, these metals turned out to have important pairwise interactions with other metals: for lead, DMA, cesium, molybdenum, and tungsten; for cadmium, cesium and uranium; for DMA, molybdenum and uranium. Cobalt was selected as the second most important variable in BART. Our AENET models suggested an inverse association between cobalt and GGT. This may not be surprising given that cobalt is an essential trace element and is part of vitamin B12 (cobalamin) which is vital to maintain human health [56]. The levels of cobalt typically found in the environment is not known to be harmful [57]. However, recent studies conducted using NHANES data found significant positive associations of cobalt with cardiovascular and cerebrovascular disease [50] and age-related impaired mobility [58]. Agarwal et al. [50] also reported a significant positive association between cardiovascular and cerebrovascular disease and tungsten which was selected as a main effect as well as interaction with lead in AENET-I and as the fourth most important variable in BART. Tungsten, commonly used in metal alloys [59], has also been positively associated with diabetes [60] and peripheral artery disease [54]. Barium, a silvery-white metal used for many different purposes in industry [61], was also selected in our multipollutant models. Urinary barium has been associated with higher insulin resistance [60], obesity [62], and lower thyroid hormone levels [63]. It should be noted that these findings are subject to reverse causality given the nature of cross-sectional design and that phenotypes such as diabetes may increase metal excretions in urine [64].

The present study has numerous limitations. We examined only metal mixtures. In real world, we are exposed to many other environmental pollutants that may cause oxidative stress leading to CVD and other health consequences. Therefore, the associations found here are still subject to potential confounding by co-pollutants other than metals. Because biomonitoring data in NHANES were not measured in all participants (i.e., some pollutants were measured only in a subset (e.g., urinary metals were measured in one third of the participants in each cycle) and different classes of pollutants were measured in different subsets in order to reduce the burden of examinations), we were not able to include other classes of pollutants in this study. Although assessment of a large number of pollutants from different classes is costly and a challenging task, it is a prerequisite for evaluating the impact of pollutant mixtures on human health.

Relatedly, the environment used in our study is limited to chemical environmental pollutants. Nonchemical stressors such as behavioral and psychosocial factors also play an important role in chronic disease risk independently as well as in conjunction with chemical pollutants. Our approach was to treat these factors as confounders, i.e., to separate the effects of nonchemical stressors out of those of the chemical pollutant mixtures. ERS can be expanded to a broader range of the environment encompassing various non-genetic factors as well as chemical-nonchemical interactions depending on investigator’s points of view and research questions. As the concept of ‘exposome’, defined as the totality of exposures over the course of a lifetime [65, 66], is emerging, our proposed approach will be useful to integrate health risks from a wide range of environmental factors identified by novel statistical approaches beyond the conventional linear regression-based modeling.

In AENET algorithms, we assumed linear (in fact, log-linear) exposure-outcome relationships and linear interactions. However, non-linear, non-monotonic dose–response relationships, for example, low-dose effects of endocrine disrupting chemicals [67], may exist. Due to such model misspecification, important variables may not be selected in AENET and not contribute to the corresponding ERS. It should be noted that the selected pairwise interactions in AENET-I may reflect non-linear dose–response relationships rather than biological interactions. In fact, in presence of non-linearity, a complex linear model attempts to capture quadratic or higher order main effect terms by estimating a non-linear interaction term [68].

In addition, for AENET and BKMR, our strategy has been to always include the covariates/confounders *Z* in the predictor space and not penalize coefficients associated with *Z*. For BART and SL we first regressed GGT on *Z* and each of the *E*’s on *Z* and used the residuals from these models as the input variables for outcome and predictors. We are aware that this does not reproduce the model *Y|E, Z*. This is driven by limitations in the implementation software. By adopting this approach, we also assume that the covariates have a linear relationship with GGT without any non-linearity or interactions within the set of *Z* and ignore metal by covariate interaction terms of the form E*Z. Future extensions can consider this possibility and attempt to treating *Z* uniformly across the methods.

The primary goal of epidemiologic research is to identify causal factors and causal structures. Although a hypothesis-driven knowledge-based approach is a standard practice to build the causal structure in epidemiologic research, an agnostic data-driven machine learning approach may be useful when limited knowledge of the causal structure is available. Many environmental chemicals, especially emerging new toxicants, have little information on underlying biological mechanisms and toxicological and epidemiological evidence that preclude us from incorporating the causal structure to the analysis. Data-driven machine learning approaches used in the present study can help us identify causal structure among environmental chemicals within a mixture. Of note, it is of less interest in public health whether the selected chemicals are causal or markers of other causal factors when those chemicals are highly correlated because regulations targeted on one chemical would likely control the other chemicals. Another important role of constructing an ERS is to study effect modification of summary exposure by other factors (for example genetic factors) and provide risk stratification in the population. For example, ERS could be used as a summary measure in a follow-up gene-environment interaction analyses or to study the effect of epigenetic changes on environmental exposure.

As mentioned earlier, we constructed an ERS for oxidative stress as a common pathway for multiple diseases to overcome the construction of multiple disease-specific ERSs. However, complex diseases such as CVD involve multiple disease pathways, and therefore, our proposed ERS may capture only partial disease risk and can be less appropriate if the selected pathway is a minor one in disease etiology. Prior knowledge regarding underlying biological mechanisms is a key prerequisite to conduct the proposed work. Ideally, multiple ERS representing each plausible biological pathway could be constructed and adaptively weighted to provide a more comprehensive risk assessment for a complex chronic disease.

Each individual metal measured in either whole blood or urine has different half-lives and therefore, may not necessarily capture the actual exposure concentrations relevant to the current disease risk. Most urinary metals used in this study have short half-lives and thus their urinary concentrations reflect exposures happened over the past hours to days. Such metals may have not been detected to be associated with GGT in our analysis and could be missed in relation to other health endpoints if long-term cumulative exposure to low levels cause those health outcomes. Another source of exposure measurement error is LODs of all metal biomarkers. For simplicity, we used a conventional ad hoc method of imputing the concentrations below LOD with LOD/√2, but it is known to result in bias especially when the proportion of concentrations below LOD is high [27]. These exposure measurement errors are generally non-differential and lead to bias towards the null if each exposure is evaluated with the outcome individually in single pollutant models. However, bias would be more complicated and even differential measurement errors may occur in multi-pollutant models when multiple pollutants have different degrees of measurement errors and those measurement errors are not independent because some of the effects of more poorly assessed pollutants may be transferred to the effect estimates of better assessed pollutants [69].

## Conclusions

In summary, the current study suggests that ERS is a useful tool for characterizing cumulative risk from pollutant mixtures, with accounting for statistical challenges such as high degrees of correlations and pollutant-pollutant interactions. ERS constructed for an intermediate marker like GGT is predictive of related disease endpoints. This new approach of multi-pollutant framework will help better understand the real-world health impacts of pollutant mixtures and facilitate risk stratification and targeted preventive intervention [20]. To overcome the methodological challenges discussed here and raised in the recent NIEHS workshop [19], collective work such as systematic assessment of a wide range of pollutants in well characterized cohorts and creation of a mixture data consortium will need to be done.

## Additional file

Additional file 1: Table S1. Supplemental tables and figures for the construction of environmental risk score beyond standard linear models using machine learning methods. (DOCX 1620 kb)

## Abbreviations

AENET-I: Adaptive elastic-net with main effects and pairwise interactions
AENET-M: Adaptive elastic-net with only main effects
As III: Arsenous acid
As V: Arsenic acid
AUC: Area under the receiver operating characteristic
BART: Bayesian additive regression tree
BKMR: Bayesian kernel machine regression
BMA: Bayesian model averaging
BMI: Body mass index
CART: Classification and regression tree
CI: Confidence interval
CVD: Cardiovascular disease
DBP: Diastolic blood pressure
DMA: Dimethylarsonic acid
ERS: Environmental risk score
GGT: Gamma-glutamyl transferase
GM: Geometric mean
GSD: Geometric standard deviation
HR: Hazard ratio
ICD: International Classification of Disease
ICP-DRC-MS: Inductively coupled-plasma dynamic reaction cell-mass spectrometry
KMR: Kernel machine regression
LASSO: Least absolute shrinkage and selection operator
LOD: Limit of detection
MCMC: Markov chain Monte Carlo
MMA: Monomethylarsonic acid
MSE: Mean squared error
MSPE: Mean squared prediction error
NHANES: National Health and Nutrition Examination Survey
NIEHS: National Institute of Environmental Health and Sciences
OR: Odds ratio
PBPK: Physiologically based pharmacokinetic
PCA: Principal component analysis
PIP: Posterior inclusion probabilities
PLS: Partial least squares
PRESS: Predicted residual sums of squares
ROC: Receiver operating characteristic
SBP: Systolic blood pressure
SD: Standard deviation
SE: Standard error
SL: Super Learner
SSE: Sum of squared errors
WQS: Weighted quantile sum

## References

[1] Braun JM, Gennings C, Hauser R, Webster TF (2016). What Can Epidemiological Studies Tell Us about the Impact of Chemical Mixtures on Human Health?. Environ Health Perspect.

[2] Chadeau-Hyam M, Campanella G, Jombart T, Bottolo L, Portengen L, Vineis P, Liquet B, Vermeulen RC (2013). Deciphering the complex: methodological overview of statistical models to derive OMICS-based biomarkers. Environ Mol Mutagen.

[3] Sun Z, Tao Y, Li S, Ferguson KK, Meeker JD, Park SK, Batterman SA, Mukherjee B (2013). Statistical strategies for constructing health risk models with multiple pollutants and their interactions: possible choices and comparisons. Environ Health.

[4] Billionnet C, Sherrill D, Annesi-Maesano I (2012). Estimating the health effects of exposure to multi-pollutant mixture. Ann Epidemiol.

[5] Tibshirani R (1996). Regression Shrinkage and Selection via the Lasso. J R Stat Soc Ser B Methodol.

[6] Zou H (2006). The Adaptive Lasso and Its Oracle Properties. J Am Stat Assoc.

[7] Zou H, Zhang HH (2009). On the Adaptive Elastic-Net with a Diverging Number of Parameters. Ann Stat.

[8] Wold S, Ruhe A, Wold H, Dunn WJ (1984). The Collinearity Problem in Linear Regression. The Partial Least Squares (PLS) Approach to Generalized Inverses. SIAM J Sci Stat Comput.

[9] Carrico C, Gennings C, Wheeler DC, Factor-Litvak P (2015). Characterization of Weighted Quantile Sum Regression for Highly Correlated Data in a Risk Analysis Setting. J Agric Biol Environ Stat.

[10] Madigan D, Raftery AE (1994). Model selection and accounting for model uncertainty in graphical models using Occam’s window. J Am Stat Assoc.

[11] Bobb JF, Valeri L, Claus Henn B, Christiani DC, Wright RO, Mazumdar M, Godleski JJ, Coull BA (2015). Bayesian kernel machine regression for estimating the health effects of multi-pollutant mixtures. Biostatistics.

[12] Breiman L, Friedman JH, Olshen RA, Stone CJ. Classification and regression trees: Wadsworth Statistics/Probability. Boca Raton: Chapman and Hall/CRC; 1984.

[13] Tin Kam H (1998). The random subspace method for constructing decision forests. IEEE Trans Pattern Anal Mach Intell.

[14] Chipman HA, George EI, McCulloch RE (2010). BART: Bayesian additive regression trees. Ann Appl Stat.

[15] Czarnota J, Gennings C, Wheeler DC (2015). Assessment of weighted quantile sum regression for modeling chemical mixtures and cancer risk. Cancer Inform.

[16] Forns J, Mandal S, Iszatt N, Polder A, Thomsen C, Lyche JL, Stigum H, Vermeulen R, Eggesbo M (2016). Novel application of statistical methods for analysis of multiple toxicants identifies DDT as a risk factor for early child behavioral problems. Environ Res.

[17] Lenters V, Portengen L, Rignell-Hydbom A, Jonsson BA, Lindh CH, Piersma AH, Toft G, Bonde JP, Heederik D, Rylander L (2016). Prenatal Phthalate, Perfluoroalkyl Acid, and Organochlorine Exposures and Term Birth Weight in Three Birth Cohorts: Multi-Pollutant Models Based on Elastic Net Regression. Environ Health Perspect.

[18] Pang Y, Peng RD, Jones MR, Francesconi KA, Goessler W, Howard BV, Umans JG, Best LG, Guallar E, Post WS (2016). Metal mixtures in urban and rural populations in the US: The Multi-Ethnic Study of Atherosclerosis and the Strong Heart Study. Environ Res.

[19] Taylor KW, Joubert BR, Braun JM, Dilworth C, Gennings C, Hauser R, Heindel JJ, Rider CV, Webster TF, Carlin DJ (2016). Statistical Approaches for Assessing Health Effects of Environmental Chemical Mixtures in Epidemiology: Lessons from an Innovative Workshop. Environ Health Perspect.

[20] Park SK, Tao Y, Meeker JD, Harlow SD, Mukherjee B (2014). Environmental Risk Score as a New Tool to Examine Multi-Pollutants in Epidemiologic Research: An Example from the NHANES Study Using Serum Lipid Levels. PLoS One.

[21] Bhatnagar A (2006). Environmental cardiology: studying mechanistic links between pollution and heart disease. Circ Res.

[22] Solenkova NV, Newman JD, Berger JS, Thurston G, Hochman JS, Lamas GA (2014). Metal pollutants and cardiovascular disease: mechanisms and consequences of exposure. Am Heart J.

[23] Ercal N, Gurer-Orhan H, Aykin-Burns N (2001). Toxic metals and oxidative stress part I: mechanisms involved in metal-induced oxidative damage. Curr Top Med Chem.

[24] Koenig G, Seneff S (2015). Gamma-Glutamyltransferase: A Predictive Biomarker of Cellular Antioxidant Inadequacy and Disease Risk. Dis Markers.

[25] Lee DH, Blomhoff R, Jacobs DR (2004). Is serum gamma glutamyltransferase a marker of oxidative stress?. Free Radic Res.

[26] van der Laan MJ, Polley EC, Hubbard AE (2007). Super Learner. Stat Appl Genet Mol Biol.

[27] Nie L, Chu H, Liu C, Cole SR, Vexler A, Schisterman EF (2010). Linear regression with an independent variable subject to a detection limit. Epidemiology.

[28] Barr DB, Wilder LC, Caudill SP, Gonzalez AJ, Needham LL, Pirkle JL (2005). Urinary creatinine concentrations in the U.S. population: implications for urinary biologic monitoring measurements. Environ Health Perspect.

[29] O’Brien KM, Upson K, Cook NR, Weinberg CR (2016). Environmental Chemicals in Urine and Blood: Improving Methods for Creatinine and Lipid Adjustment. Environ Health Perspect.

[30] Yang Y, Zou H (2015). Package ‘gcdnet’.

[31] Chipman H, McCulloch R (2016). Package ‘BayesTree’.

[32] Kapelner A, Bleich J (2016). Package ‘bartMachine’.

[33] Bobb JF (2017). Package ‘bkmr’.

[34] Polley E, LeDell E, Kennedy C, Lendle S, van der Laan M (2016). Package ‘SuperLearner’.

[35] Thiebaut AC, Benichou J (2004). Choice of time-scale in Cox’s model analysis of epidemiologic cohort data: a simulation study. Stat Med.

[36] Khoury MJ, Wacholder S (2009). Invited commentary: from genome-wide association studies to gene-environment-wide interaction studies--challenges and opportunities. Am J Epidemiol.

[37] Draper NR, Vannostrand RC (1979). Ridge Regression and James-Stein Estimation - Review and Comments. Technometrics.

[38] Yuan M, Lin Y (2006). Model selection and estimation in regression with grouped variables. J R Stat Soc Ser B Stat Methodol.

[39] Shmueli G (2010). To explain or to predict?. Stat Sci.

[40] Bühlmann P, van de Geer S. Statistics for High-Dimensional Data: Methods, Theory and Applications. Heidelberg: Springer; 2011.

[41] Bradley RD, Fitzpatrick AL, Jacobs DR, Lee DH, Swords Jenny N, Herrington D (2014). Associations between gamma-glutamyltransferase (GGT) and biomarkers of atherosclerosis: the Multi-ethnic Study of Atherosclerosis (MESA). Atherosclerosis.

[42] Janicki-Deverts D, Cohen S, Matthews KA, Gross MD, Jacobs DR (2009). Socioeconomic status, antioxidant micronutrients, and correlates of oxidative damage: the Coronary Artery Risk Development in Young Adults (CARDIA) study. Psychosom Med.

[43] Van Hemelrijck M, Jassem W, Walldius G, Fentiman IS, Hammar N, Lambe M, Garmo H, Jungner I, Holmberg L (2011). Gamma-glutamyltransferase and risk of cancer in a cohort of 545,460 persons - the Swedish AMORIS study. Eur J Cancer.

[44] Milne GL, Musiek ES, Morrow JD (2005). F2-isoprostanes as markers of oxidative stress in vivo: an overview. Biomarkers.

[45] Roberts LJ, Morrow JD (2000). Measurement of F(2)-isoprostanes as an index of oxidative stress in vivo. Free Radic Biol Med.

[46] NTP (2012). NTP monograph on health effects of low-level lead. NTP Monogr.

[47] Moon K, Guallar E, Navas-Acien A (2012). Arsenic exposure and cardiovascular disease: an updated systematic review. Curr Atheroscler Rep.

[48] Navas-Acien A, Sharrett AR, Silbergeld EK, Schwartz BS, Nachman KE, Burke TA, Guallar E (2005). Arsenic exposure and cardiovascular disease: a systematic review of the epidemiologic evidence. Am J Epidemiol.

[49] Tellez-Plaza M, Jones MR, Dominguez-Lucas A, Guallar E, Navas-Acien A (2013). Cadmium exposure and clinical cardiovascular disease: a systematic review. Curr Atheroscler Rep.

[50] Agarwal S, Zaman T, Tuzcu EM, Kapadia SR (2011). Heavy metals and cardiovascular disease: results from the National Health and Nutrition Examination Survey (NHANES) 1999–2006. Angiology.

[51] Guo J, Su L, Zhao X, Xu Z, Chen G (2016). Relationships between urinary antimony levels and both mortalities and prevalence of cancers and heart diseases in general US population, NHANES 1999–2010. Sci Total Environ.

[52] Lind PM, Olsen L, Lind L (2012). Circulating levels of metals are related to carotid atherosclerosis in elderly. Sci Total Environ.

[53] Mendy A, Gasana J, Vieira ER (2012). Urinary heavy metals and associated medical conditions in the US adult population. Int J Environ Health Res.

[54] Navas-Acien A, Silbergeld EK, Sharrett R, Calderon-Aranda E, Selvin E, Guallar E (2005). Metals in urine and peripheral arterial disease. Environ Health Perspect.

[55] Nigra AE, Ruiz-Hernandez A, Redon J, Navas-Acien A, Tellez-Plaza M (2016). Environmental Metals and Cardiovascular Disease in Adults: A Systematic Review Beyond Lead and Cadmium. Curr Environ Health Rep.

[56] Lindsay D, Kerr W (2011). Cobalt close-up. Nat Chem.

[57] ATSDR (2004). Toxicological profile for cobalt.

[58] Lang IA, Scarlett A, Guralnik JM, Depledge MH, Melzer D, Galloway TS (2009). Age-related impairments of mobility associated with cobalt and other heavy metals: data from NHANES 1999–2004. J Toxicol Environ Health A.

[59] Keith LS, Wohlers DW, Moffett DB, Rosemond ZA (2007). ATSDR evaluation of potential for human exposure to tungsten. Toxicol Ind Health.

[60] Menke A, Guallar E, Cowie CC (2016). Metals in Urine and Diabetes in U.S. Adults. Diabetes.

[61] ATSDR (2007). Toxicological profile for barium.

[62] Padilla MA, Elobeid M, Ruden DM, Allison DB (2010). An examination of the association of selected toxic metals with total and central obesity indices: NHANES 99–02. Int J Environ Res Public Health.

[63] Yorita Christensen KL (2013). Metals in blood and urine, and thyroid function among adults in the United States 2007–2008. Int J Hyg Environ Health.

[64] Chaumont A, Nickmilder M, Dumont X, Lundh T, Skerfving S, Bernard A (2012). Associations between proteins and heavy metals in urine at low environmental exposures: evidence of reverse causality. Toxicol Lett.

[65] Wild CP (2005). Complementing the genome with an “exposome”: the outstanding challenge of environmental exposure measurement in molecular epidemiology. Cancer Epidemiol Biomark Prev.

[66] Wild CP (2012). The exposome: from concept to utility. Int J Epidemiol.

[67] Rhomberg LR, Goodman JE (2012). Low-dose effects and nonmonotonic dose-responses of endocrine disrupting chemicals: has the case been made?. Regul Toxicol Pharmacol.

[68] Sofer T, Cornelis MC, Kraft P, Tchetgen Tchetgen EJ (2017). Control Function Assisted Ipw Estimation with a Secondary Outcome in Case–control Studies. Stat Sin.

[69] Zeger SL, Thomas D, Dominici F, Samet JM, Schwartz J, Dockery D, Cohen A (2000). Exposure measurement error in time-series studies of air pollution: concepts and consequences. Environ Health Perspect.

