# Supplementary material for: Construction of environmental risk score beyond standard linear models using machine learning methods: application to metal mixtures, oxidative stress and cardiovascular disease in NHANES
Source: Environ Health. 2017 Sep 26;16:102. doi: 10.1186/s12940-017-0310-9 (PMC5615812; doi:10.1186/s12940-017-0310-9)
Supplement: Additional file 1: Table S1. — Supplemental tables and figures for the construction of environmental risk score beyond standard linear models using machine learning methods. (DOCX 1620 kb) [file 12940_2017_310_MOESM1_ESM.docx]

**Additional File 1. Supplemental tables and figures for the construction of environmental risk score beyond standard linear models using machine learning methods.**

Table S1. Characteristics of the participants included and excluded.

|  | Population Included | Population Excluded | P Value | |
| --- | --- | --- | --- | --- |
|  | N=9664 | N=1141 | |  |
| CONTINUOUS, mean(SD) | |  | |  |
| Age, years | 49.2 (17.2) | 52.6 (19.7) | | 3.09E-09 |
| BMI, kg/m^2^ | 28.8 (6.69) | 29.0 (8.01) | | 6.47E-02 |
| Creatinine, µmol/L | 121.0 (78.08) | 131.1 (88.93) | | 2.30E-04 |
| CATEGORICAL, N (%) |  |  | |  |
| Female | 4911 (50.82) | 629 (55.13) | | 6.47E-03 |
| Race/Ethnicity |  |  | | 2.62E-10 |
| Mexican American | 1618 (16.74) | 143 (12.53) | |  |
| Other Hispanic | 779 (8.06) | 96 (8.41) | |  |
| Non-Hispanic White | 4501 (46.57) | 477 (41.81) | |  |
| Non-Hispanic Black | 1976 (20.45) | 330 (28.92) | |  |
| Other | 790 (8.17) | 95 (8.33) | |  |
| Smoking Status |  |  | | 1.35E-01 |
| Never | 5242 (54.24) | 608 (53.43) | |  |
| Former | 2000 (20.70) | 263 (23.46) | |  |
| Current | 2422 (25.06) | 267 (23.11) | |  |
| Education |  |  | | 5.73E-04 |
| <High School | 2599 (26.89) | 352 (31.18) | |  |
| High School | 5018 (51.92) | 584 (51.73) | |  |
| College or Above | 2047 (21.18) | 193 (17.09) | |  |

Table S2. Summary of variable selection results for gamma-glutamyl transferase (GGT) in adaptive elastic net-main effects (AENET-M) and adaptive elastic net-interactions (AENET-I).

|  | AENET-M | | AENET-I | |
| --- | --- | --- | --- | --- |
|  | β (SE) | p-value | β (SE) | p-value |
| Lead* | 0.051 (0.011) | 5.95E-06 | 0.311 (0.069) | 6.24E-06 |
| Mercury* | 0.003 (0.006) | 6.30E-01 | -0.057 (0.021) | 5.47E-03 |
| DMA | 0.061 (0.011) | 4.65E-08 | 0.278 (0.069) | 5.93E-05 |
| Barium | 0.041 (0.008) | 1.20E-07 | 0.048 (0.008) | 7.91E-10 |
| Cadmium | 0.074 (0.010) | 9.95E-15 | 0.222 (0.045) | 9.84E-07 |
| Cobalt | -0.086 (0.011) | 7.51E-15 | -0.139 (0.024) | 1.20E-08 |
| Cesium | -0.081 (0.015) | 4.34E-08 | -0.062 (0.023) | 5.89E-03 |
| Molybdenum | -0.045 (0.010) | 3.92E-06 | -0.011 (0.017) | 5.09E-01 |
| Uranium | 0.041 (0.008) | 5.91E-08 | 0.041 (0.021) | 5.38E-02 |
| Lead |  |  | -0.084 (0.016) | 1.20E-07 |
| MMA |  |  | -0.097 (0.015) | 1.34E-10 |
| As III |  |  | 0.045 (0.012) | 1.91E-04 |
| Tungsten |  |  | 0.013 (0.009) | 1.32E-01 |
| Lead*×DMA |  |  | 0.086 (0.034) | 1.03E-02 |
| Lead*×Cesium |  |  | -0.145 (0.041) | 4.54E-04 |
| Lead*×Molybdenum |  |  | -0.05 (0.032) | 1.29E-01 |
| Lead*×Tungsten |  |  | 0.062 (0.026) | 1.68E-02 |
| Mercury*×As V |  |  | -0.152 (0.081) | 6.09E-02 |
| Mercury*×Cesium |  |  | 0.053 (0.020) | 8.98E-03 |
| DMA×Molybdenum |  |  | -0.048 (0.023) | 3.48E-02 |
| DMA×Uranium |  |  | 0.058 (0.021) | 6.20E-03 |
| Cadmium×Cesium |  |  | -0.023 (0.022) | 3.00E-01 |
| Cadmium×Uranium |  |  | 0.057 (0.017) | 7.69E-04 |
| Cobalt×Antimony |  |  | -0.041 (0.018) | 2.16E-02 |

Asterisk next to the metal names indicates metals measured in whole blood. As, arsenic; As III, arsenous acid; As V, arsenic acid; MMA, monomethylarsonic acid (MMA); DMA, dimethylarsonic acid.

Table S3. Summary of variable importance measures in BART and SL and their rank orders and the sum of the rank orders between BART and SL.

|  | Variable Importance | |  | Rank | | |
| --- | --- | --- | --- | --- | --- | --- |
|  | BART (%)^a^ | SL (%)^b^ |  | BART | SL | Sum |
| Cadmium | 9.49 | 21.5 |  | 1 | 6 | 7 |
| Tungsten | 7.13 | 22.9 |  | 4 | 3 | 7 |
| MMA | 6.78 | 24.2 |  | 5 | 2 | 7 |
| DMA | 7.14 | 22.6 |  | 3 | 5 | 8 |
| As V | 4.83 | 25.1 |  | 9 | 1 | 10 |
| Barium | 5.67 | 21.2 |  | 7 | 7 | 14 |
| Cobalt | 7.21 | 15.2 |  | 2 | 14 | 16 |
| Molybdenum | 5.02 | 18.6 |  | 8 | 11 | 19 |
| Total As | 3.66 | 22.6 |  | 16 | 4 | 20 |
| Lead* | 5.68 | 12.0 |  | 6 | 16 | 22 |
| Lead | 4.20 | 19.1 |  | 12 | 10 | 22 |
| Antimony | 3.42 | 20.9 |  | 17 | 8 | 25 |
| Thallium | 3.39 | 20.5 |  | 18 | 9 | 27 |
| Uranium | 4.72 | 9.4 |  | 10 | 18 | 28 |
| Cadmium* | 3.96 | 13.4 |  | 13 | 15 | 28 |
| AsC | 3.78 | 15.6 |  | 15 | 13 | 28 |
| Cesium | 4.41 | 1.3 |  | 11 | 20 | 31 |
| As III | 3.86 | 9.9 |  | 14 | 17 | 31 |
| AsB | 2.68 | 17.9 |  | 20 | 12 | 32 |
| Mercury* | 2.98 | 8.4 |  | 19 | 19 | 38 |

BART, Bayesian Additive Regression Tree; SL, Super Learner. As, arsenic; As III, arsenous acid; As V, arsenic acid; MMA, monomethylarsonic acid (MMA); DMA, dimethylarsonic acid. Asterisk next to the metal names indicates metals measured in whole blood.

^a^The variable importance for BART corresponds to the relative proportion of the contribution of each specific metal to the aggregate classification prediction.

^b^The variable importance for SL was computed as the difference between the sum of squared error (SSE) of the SL with all 20 metals and the SSE with the SL without each corresponding metal [SSE(-i)] divided by SSE [i.e., (SSE(-i) – SSE)/SSE].

Table S4. Comparison of ERS distribution and risk prediction performance by individual algorithms within SL and SL.

|  | random forest | glm interaction | ridge | glmnet | SVM | SL |
| --- | --- | --- | --- | --- | --- | --- |
| Distributions of ERS | | | | | | |
| Training Set |  |  |  |  |  |  |
| Mean (SD) | 0.00 (0.16) | 0.00 (0.08) | 0.00 (0.04) | 0.00 (0.04) | 0.00 (0.07) | 0.00 (0.06) |
| Range | (-0.38, 0.94) | (-0.39, 0.85) | (-0.18, 0.17) | (-0.18, 0.16) | (-0.33, 0.31) | (-0.18, 0.27) |
|  |  |  |  |  |  |  |
| Testing Set |  |  |  |  |  |  |
| Mean (SD) | 0.01 (0.05) | 0.00 (0.07) | 0.00 (0.04) | 0.00 (0.04) | -0.02 (0.06) | 0.00 (0.04) |
| Range | (-0.15, 0.41) | (-0.46, 0.38) | (-0.20, 0.18) | (-0.20, 0.17) | (-0.23, 0.27) | (-0.17, 0.24) |
|  |  |  |  |  |  |  |
| Risk Prediction Performance | | | | | | |
| Continuous GGT^c^ | | | | | | |
| Training Set |  |  |  |  |  |  |
| Correlation^d^ | 0.92 | 0.30 | 0.22 | 0.23 | 0.47 | 0.75 |
| MSE | 1.5E-03 | 6.6E-02 | 7.0E-02 | 7.0E-02 | 5.5E-02 | 3.6E-02 |
|  |  |  |  |  |  |  |
| Testing Set |  |  |  |  |  |  |
| Correlation^d^ | 0.18 | 0.18 | 0.25 | 0.25 | 0.17 | 0.26 |
| PRESS | 325.4 | 326.1 | 319.7 | 319.5 | 325.9 | 321.7 |
| MSPE | 6.7E-02 | 6.7E-02 | 6.6E-02 | 6.6E-02 | 6.7E-02 | 6.6E-02 |
|  | | | | | | |
| Dichotomous GGT^e^ | | | | | | |
| Training Set |  |  |  |  |  |  |
| AUC | >0.99 | 0.74 | 0.70 | 0.70 | 0.80 | 0.92^‡^ |
| 95% CI | (0.99, 1.00) | (0.72, 0.76) | (0.67, 0.72) | (0.67, 0.72) | (0.78, 0.82) | (0.91, 0.93) |
|  |  |  |  |  |  |  |
| Testing Set |  |  |  |  |  |  |
| AUC | 0.69 | 0.68 | 0.70 | 0.70 | 0.68 | 0.70^*^ |
| 95% CI | (0.66, 0.71) | (0.66, 0.71) | (0.67, 0.72) | (0.67, 0.72) | (0.66, 0.70) | (0.67, 0.72) |

^a^Base model contains only covariates (age, sex, race/ethnicity, smoking status, education, body mass index, urinary creatinine).

^b^Full model contains all covariates, main effects and all possible pairwise interactions of metals.

^c^GGT was logarithmically transformed. Mean (SD) of log(GGT) = 0.27 (0.21).

^d^Correlation between GGT and ERS.

^e^GGT was dichotomized at the 90^th^ percentile (50 I/U).

^*^*P*<0.1, ^†^*P*<0.05, ^‡^*P*<0.01. *P*-values were computed with permutation tests comparing with AUC of the base model.


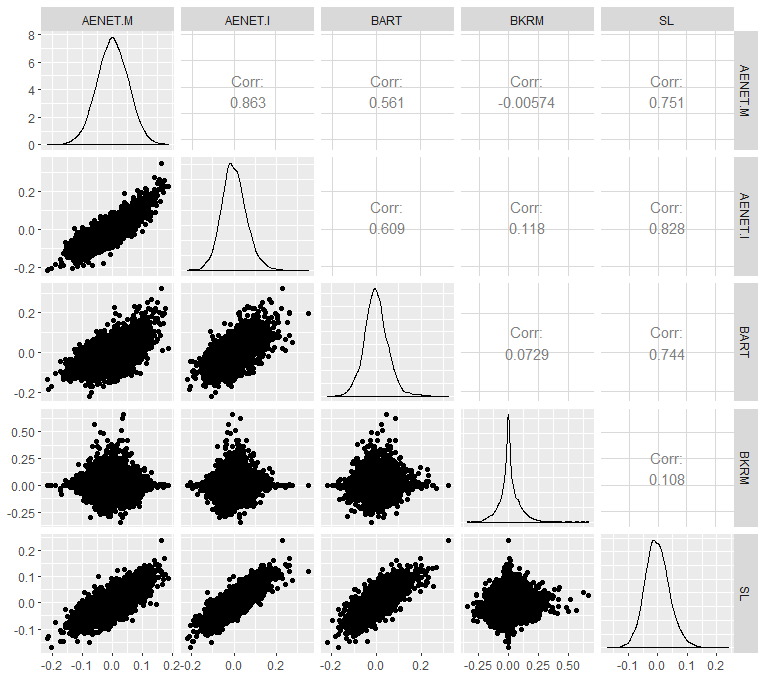


Figure S1. Pearson correlation plots between ERS’s. AENET-M, adaptive elastic-net with main effects only; AENET-I, adaptive elastic-net with main effects and pairwise interactions; BART, Bayesian additive regression tree; BKMR, Bayesian kernel machine regression; SL, Super Learner.

**
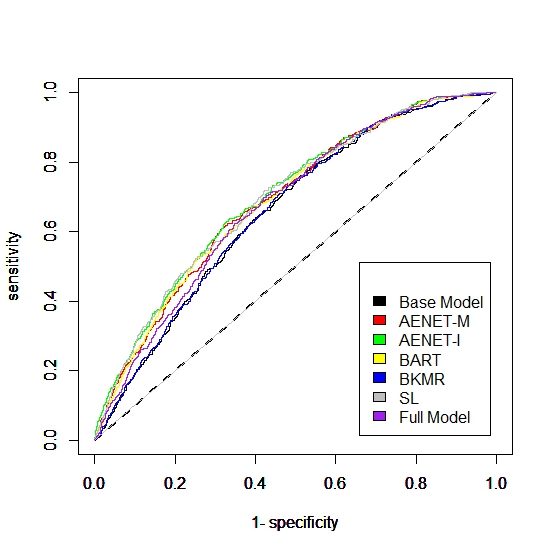
**

Figure S2. Receiver operating characteristic (ROC) curves for gamma-glutamyl transferase (GGT) for different ERS models. The dotted line denotes the null curve. The base model (black) is for the model with only covariates. The full model (purple) is for the model with all main effects and their pairwise interactions (saturated model). AENET-M, adaptive elastic-net with main effects only; AENET-I, adaptive elastic-net with main effects and pairwise interactions; BART, Bayesian additive regression tree; BKMR, Bayesian kernel machine regression; SL, Super Learner.
